# How to integrate physiological data from wearables in treatment of personality disorders: a narrative review

**DOI:** 10.3389/fpsyt.2025.1591871

**Published:** 2025-06-18

**Authors:** Luuk L. Lans, Klaas M. L. Huijbregts, Gerben J. Westerhof, Suzanne W. Haeyen, Youri P. M. J. Derks, Matthijs L. Noordzij

**Affiliations:** ^1^ Personality Disorders, Mental Health Care Facility Department of Ggnet/Scelta, Warnsveld, Netherlands; ^2^ Department of Psychology, Health and Technology, University of Twente, Enschede, Netherlands; ^3^ Department Arts Therapies, HAN University of Applied Sciences, Nijmegen, Netherlands

**Keywords:** BPD, borderline personality disorder, biocueing, biofeedback, interoception, wearables

## Abstract

In recent years, stress-monitoring innovations using wearable technology have entered the market. One innovation is biocueing, a process where patients receive real-time feedback on passive monitoring of significant changes in their physiological data, such as (additional) heart rate, heart rate variability or skin conductance. This technology offers potential for patients with borderline personality disorder, as they often report severe stress, difficulties in emotion regulation and low levels of emotional- and body awareness. Yet, currently there is no clear direction on when and how to fit these technologies, and physiology in general, into treatments for borderline personality disorder. We provide a comprehensive review on how and to what extent evidence-based treatments (Transference Focused Psychotherapy, Mentalization Based Treatment, Schema Therapy, Dialectical Behavior Therapy), and their underpinning theories provide guidance and predictions for integrating these technologies. Only Dialectical Behavior Therapy provide a theoretical framework that includes physiology, as well as interventions that actively target physiological data, whereas the other evidence-based treatments largely disregard physiology. Other promising developments are Creative Arts and Psychomotor Therapies and the Polyvagal theory, as they target bodily sensations and physiology more directly. Four avenues for future research and integration of psychophysiological theory and wearable technology in treatment are discussed: abandoning physiological data and technology, keeping a human in the loop, machine-learning biocueing interventions, or biomonitoring devices as long-term (mental) health monitors.

## Background

Borderline personality disorder (BPD) is characterized by emotional dysregulation, impulsivity, and intense interpersonal conflicts (1), making it a candidate for exploring the potential of adding biocueing technology to treatment. Biocueing, an innovation of biofeedback, involves wearables such as smartwatches, smart rings, or smart clothing that provide cues about physiological changes in daily life (2). The technology is hypothesized to target interoceptive processes by increasing awareness of physiological processes, with aims of contributing to emotion regulation. Nonetheless, the field of biocueing is still in its infancy, raising many questions about its intended purpose, underlying mechanisms, practicality, and ethical considerations. Biocueing technologies, however, are emerging (e.g (3). and already used by the general population in the domain of stress management (4). This underscores the need to gain insights on their potential. In this narrative review we discuss and examine (I) the state-of-the-art biocueing technologies, (II) how physiology relates to treatments of borderline personality disorder, (III) whether the theories underpinning the treatments are in line with latest psychophysiological insights, (IV) directions for future research on the integration of biocueing technologies.

Wearables are nowadays widely accepted, unobtrusively worn, and non-invasive. Most modern wearables can measure continuous physiological data (5–7), including heart rate (HR), heart rate variability (HRV), breathing rate, or skin conductance in a naturalistic environment (8). The reliability and validity of these devices have been questionable (9) but are improving and offer a trade-off between noise (substantial) and quantity of data (also substantial) thanks to the longitudinal recording these devices allow (10). Part of these physiological data relate to emotions (11, 12). For instance, heart rate and breathing increase rapidly in response to a threat preparing the entire organism for adaptive responses (13–15). This physiological activation is intertwined with emotions, which are not merely bodily reactions but also involve subjective experience and behavior. Emotions emerge from the dynamic interplay between the body, psychological experience, and environment, shaping and being shaped by social and contextual factors (16). To further complicate matters, various other variables also impact one’s physiology, not in the least physical activity, sleep deprivation, coffee and alcohol consumption, cardioactive medication, age, gender, and many more (17). Therefore, it is not surprising it has been challenging to find biomarkers that can accurately capture emotion dynamics. Some biomarkers, such as HRV or respiratory sinus arrhythmia (RSA; a measure of synchronization of heart rate and respiratory), are frequently regarded as reliable biomarkers for measuring emotion regulation and stress (15, 18, 19). With ongoing development in the field of wearables, it is expected to further develop in the coming years (20).

However, this is a field of debate where some are looking for biomarkers or ‘emotion fingerprints’, while others emphasize the context sensitivity and absence of a 1-to-1 mapping between emotions and physiological responses. A meta-analytic investigation has demonstrated that there is no direct mapping between an emotion category and a specific autonomic nervous system response pattern (21). Additionally, there is substantial variability in autonomic nervous system changes during instances of the same emotion category, which is not accounted for by experimental moderators such as the method of emotion induction. These findings suggest that autonomic nervous system changes during emotion are more akin to a population of dynamic, context-sensitive- and individual circumstances rather than a consistent bodily fingerprint. These insights emphasize the importance of considering the context and variability of physiological responses, personalized models of physiology and strongly suggest that biocueing technologies must be adaptable and context-aware (or have a human in the loop to assess context) to be effective in clinical practice.

Biocueing combines passive mobile sensing with just-in-time adaptive interventions (JITAI) (2, 22, 23). The field of JITAI interventions is emerging in psychological research and entails interventions that are tailored in-the-moment, when regulation or support is most needed (24, 25). Current biocueing technologies deliver interventions ranging from identifying physiological stress levels to explicit interventions such as breathing exercises, meditation, mindfulness or biofeedback exercises (2). For instance, Leonard and colleagues (23), employed an electrodermal activity (EDA) sensor band for passive monitoring and applied certain cognitive behavioral exercises as JITAI interventions. Reimer et al. (26) developed an application uses HRV for calibrating individual stress levels, to intervene by signaling and providing biofeedback exercises. Yet others developed a mobile application that combines passive heart rate sensing on a smartwatch with vibrations on heightened physiological reactivity (27). Whereas current studies often use predetermined JITAI strategies, it could also be compelling to personalize interventions or use cues for behavioral experiments. Further research is needed on different types of interventions.

One group of individuals that could benefit from biocueing technology are individuals with borderline personality disorders (BPD). There is a consensus that emotional dysregulation is a core characteristic of BPD (e.g. (28). These individuals lack adaptive emotion regulation strategies (29), seek relief in non-suicidal self-injury behaviors (30) and occasionally need to be admitted for psychiatric hospitalizations (31). In the last decades, many different psychotherapies for BPD have developed that garnered support in one or more randomized controlled trials (32). According to the Dutch Multidisciplinary Guidelines for personality disorders, four evidence-based psychotherapeutic treatments are to be considered (33): Dialectical Behavior Therapy (DBT) (34), Mentalization Based Treatment (MBT) (35), Transference Focused Psychotherapy (TFP) (36), and, Schema Therapy (ST) (37), with optional additional treatments such as Creative Arts and Psychomotor Therapies (CAPTs), pharmacotherapy, social-psychiatric or systemic interventions. Each of the evidence-based psychotherapeutic treatments for BPD takes several months to years to complete and are costly for society (38). Although the effectiveness of these BPD treatments slightly outperforms treatment as usual, their effectiveness is small to moderate at best (39). This underscores our need for alternative and innovative approaches and interventions that target emotion regulation.

Unfortunately, adaptive emotion regulation skills are not easily attained. There is no one-size-fits-all approach to emotion regulation. According to Linehan’s biosocial theory, for example, emotion regulation is a capacity that emerges during childhood in interaction with significant others (34). Gross, in another line of research, has extensively studied the concept of emotion regulation itself. He defines emotion regulation as a dynamic interplay of identification, appraisal, and action, with the goal of modulating emotional responses (40). Gross underscores the importance of the identification stage, particularly emotional awareness, as a precursor for selecting adaptive emotion regulation strategies (41, 42). Of course, the capacity to consciously experience and identify emotions in a fine-grained manner is advantageous for selecting suitable emotion regulation strategies (43). For instance, individuals with low levels of emotional awareness fail to notice bodily changes and struggle to verbalize emotional states (44). Thus, it is unsurprising that emotional awareness and interoception are tightly linked (45–47). Interoception is defined by (48) as ‘all top-down and bottom-up processes by which an organism senses, interprets, and integrates signals from within itself and below the skin, across conscious and nonconscious levels’.

The field of interoception has been in motion in the last decade. Since 2015, Garfinkel et al. subdivided the concept of interoception into three distinct components: *interoceptive accuracy* (objective accuracy of internal signals using standardized procedures like heartbeat detection task or heartbeat counting task), *interoceptive sensibility* (self-rated perception of internal signals, e.g. questionnaires), and *interoceptive awareness* (discrepancies between interoceptive accuracy and interoceptive sensibility) (49). This distinction is relevant to address the paradox of attempting to objectively assess a phenomenon that is, by its very nature, experienced subjectively. Indeed, both objective and subjective measures have their respective advantages and weaknesses. Although improving interoceptive accuracy may be helpful for the identification of emotions, it is debatable whether interventions should target interoceptive accuracy alone and by itself. On the one hand, Brener et al. found that only 30% of the general population pass the interoceptive cardiac accuracy tests, suggesting that perceptual accuracy of one’s heartbeat should not be considered the norm (50). On the other hand, subjective measures face the challenge of self-report, which implies that individuals need to be self-aware of their interoceptive deficiencies in order to be able to report them.

Individuals with BPD frequently report low levels of emotional awareness in comparison to healthy controls (51). With regard to interoception, alterations in interoceptive processes are reported by individuals with BPD (52). The most compelling evidence derives from two studies that report alterations in the assessment of objective physiological states using heartbeat evoked potentials, a method using electroencephalogram (EEG) to measure cortical processing of cardiac signals (53, 54). According to Back et al., these results suggest deficits in afferent and efferent information processing along the brain-body axis in patients with BPD (52). Furthermore, alterations in interoceptive sensibility are found in three studies, wherein individuals with BPD show more disregard and dissociation from body sensations on self-report questionnaires (55–57). Alterations were not, or not consistently, reported on interoceptive accuracy (56, 58, 59) and interoceptive awareness (56). Collectively, these findings suggest the presence of some alterations in interoceptive processing, predominantly based on neural approaches and subjective beliefs about body perception and body dissociation.

With the aid of technology, it is now possible to facilitate one’s awareness of objective physiological processes and potentially boost different aspects of interoception. Nevertheless, the applicability and efficacy of these technologies remain uncertain. For example, a recent review revealed that HRV biofeedback therapy yielded inconsistent results in improving interoceptive accuracy (60). One of their recommendations is a higher intensity and number of sessions, to increase the probability for interoceptive change (60). It is plausible that biocueing aligns with this recommendation, providing that wearables allow for ongoing measurements and feedback in real-time and in relevant daily life situations. Yet, the effectiveness of biocueing remains speculative. An initial review of ter Harmsel et al. (2021) included 26 ambulatory biofeedback and four biocueing studies, and found that the use of biocueing resulted in better capabilities to verbalize emotions, increased self-control, and anger management (61). Another review, reported that the utilization of sensing wearables is sufficiently acceptable and feasible, and advises paying attention to the transition phase toward implementation. They underscore the need for embedding sensing technology in clinical protocols and treatments. Thus, to successfully implement biocueing in treatments for borderline personality disorder, it is necessary to align it with the therapeutic frames (DBT, TFP, MBT, ST, CAPTs) and their underpinning theories.

## Methods

This narrative review has two main objectives: (I) to examine how different treatment modalities and theories describe and utilize interoception and physiology within their treatment, and (II), to examine if these treatments and underpinning theories are consistent with recent psychophysiological literature.

Firstly, this review was initiated with an examination of key treatment manuals for PDs, including Dialectical Behavior Therapy (DBT) (34), Mentalization Based Treatment (MBT) (35), Transference Focused Psychotherapy (TFP) (36), and, Schema Therapy (ST) (37). The main focus was to identify how different treatments conceptualize, utilize and incorporate physiological aspects within their treatment. Secondly, a non-systematic literature search was conducted between November 2023 to November 2024 in Pubmed and Scholar. The search strategy covered combinations of “physiology” [e.g. physiology, psychophysiology] with “therapeutic frames [e.g. DBT/MBT/TFP/ST]”, combinations of “physiology” with “underpinning theories [e.g. Attachment, Biosocial theory, Polyvagal theory]”, and combinations of “biomarkers [e.g. HRV, HR, SCR]” with “therapeutic frames [DBT/MBT/TFP/ST]”. Studies were included when they examined either psychophysiological or autonomic processes within evidence-based treatments for PDs, or interventions or technologies that utilize physiology within the treatment of PDs. The present study excluded alternative psychotherapeutic treatments for personality disorders such as General Psychiatric Management, Client-Centred Psychotherapy and Body-Focused Treatments, as well as more generic forms of psychotherapy. After examination, those studies that use related terms to autonomic processes without any explicit reference to physiological processes (e.g. arousal or tension) were also excluded. Finally, the search was expanded through cross-referencing and a broad scan of the grey literature. As the grey literature can be vast, it was explored for relevant sources on the intersection of (psycho)physiology, biofeedback and personality disorders. While a systematic quality assessment was not conducted, preferences was given to higher-level evidence such as review studies and, when available, meta-analysis.

### Personality disorder treatments and their underpinning theories

For each section, we start with a brief description of the treatment and its underlying theory, followed by a summary of physiology is integrated within the treatment model, and end with a reflection on psychophysiological literature.

### Dialectical behavior therapy

#### Brief overview of the treatment and its underlying theory

DBT is a structured cognitive behavioral treatment, developed by Linehan, targeting suicidal behaviors and improving emotional dysregulation (34). The standard Dialectical Behavior Therapy (DBT) program comprises weekly individual therapy sessions, group skills training sessions, and an optional 24-hour consultation service. This core program may be further augmented with complementary interventions such as Creative Arts Therapy and Psychomotor Therapy (for further details on these interventions and their physiological underpinnings, see **Box 1**). DBT offer a wide variety of emotion regulation skills which requires sufficient practice so that they ultimately become automatic. These skills include self-monitoring, reappraisals, self-awareness and acceptance skills. The duration of treatment typically spans six to twelve months, although longer interventions are common for individuals with more severe symptomatology (62). The treatment is based upon Linehan’s Biosocial Theory, a theory that posits that emotion regulation deficits result from a biological predisposition with higher physiological baseline values (hypersensitivity), hyperreactivity to emotional events, and an impaired habituation with a slower return to baseline levels (34). The combination of a biological predisposition and adverse childhood experiences culminates in difficulties in labeling, expressing, and modulating emotions.

Box 1 **Creative arts and psychomotor therapies (CAPTs).**

Alongside the first recommended evidence-based treatments, other interventions are Creative Arts and Psychomotor Therapies (CAPTs). According to the Dutch Multidisciplinary Guidelines for PD, CAPTs are advised with goals of getting into contact with difficult aspects of the functioning PD patients and their experiences, to work on goals such as regulation of emotions, stress, identity/self-image, self-expression, mood/anxiety, relaxation, changing patterns and social functioning (33, 76).

Creative Arts and Psychomotor Therapies (CAPTs) include arts-, drama-, music-, dance-, and psychomotor therapy. CAPTs have an experiential, action-directed and creative quality, are body and movement oriented and make methodical and targeted use of a wide range of working methods, materials, instruments and attributes. Although there is a general consensus of which interventions belong to which CAPTs, treatment protocols are of limited use in scientific studies. Different CAPTs have not one unique guidebook, but rather multiple distinct treatment manuals available. The objective of the present study did not include an investigation of these different guidebooks to scan for potential interventions that engage with physiology. Broadly speaking, it can be said that Psychomotor and Dance Therapy aim to improve mental health through movement, physical activity, yoga, posture, and body awareness exercises (77). Music, Dance, Drama and Visual Art Therapy make use of artistic expression, material interaction, body awareness and playfulness interventions. The CAPTs are working towards a clear underpinning theory, among which the polyvagal theory is increasingly regarded as a potential candidate [e.g (78). or (79)].

How does psycho(physiological) literature support CAPTs?

Although the research field of CAPTs has been expanding significantly in recent years, psychophysiological research on CAPTs in psychiatry remains relatively limited. To the best of our knowledge, no study has used psychophysiological measures during CAPTs focused on patients with PDs or BPD. This may be attributed, at least in part, to the fact that a research culture is still relatively underdeveloped in this field. Nevertheless, preliminary evidence in other target groups suggests that specific CAPTs interventions may prove beneficial for psychophysiological outcomes. For example, a comprehensive review of HRV and yoga therapy demonstrated that frequent yoga-practitioners exhibit higher baseline HRV compared to non-frequent yoga practitioners (80). However, it is difficult to interpret these results, given the relatively low quality of the studies included and potential mediator factors. Another systematic review shows that brief music therapy may have favorable effects on HR, blood pressure, and HRV (81). Yet, the majority of these studies were conducted in a hospital setting which limits the extent to which their findings can be applied to psychiatry. Nevertheless, it may be worthwhile to explore if listening to one’s own reference music may be a beneficial JITAI intervention for BPD.

In sum, it could be promising to further explore if biocueing interventions could align CAPTs, given their innate bottom-up approach working from bodily sensations. However, as is often the case, there is a need for more studies that makes use of psychophysiological measures during CAPTs, particularly within the field of psychiatry.

#### Utilization of physiology in their treatment model

In her DBT skill manual, Linehan integrates physiology into a variety of intervention strategies. For one, individuals engaged in DBT undertake daily self-monitoring, assessing difficult situations in terms of perceived emotional states, thoughts, behaviors, and physiological reactions. This facilitates bodily awareness by fostering an understanding of the ways the body responds to certain triggers. Another central component of DBT are mindfulness and relaxation exercises, which are designed to improve both awareness and promote the relaxation of the autonomic nervous system. Finally, DBT provides a comprehensive array of emotion-regulation skills, including the TIPP interventions (acronym of interventions) for individuals experiencing heightened physiological arousal: Tip the temperature (e.g. taking a cold shower), Intensive exercise, Paced breathing (5–6 breaths per minute), and Progressive muscle relaxation. Finally, Linehan elaborates on specific interventions in which she integrates physiological sensations. For example, in one intervention, she combines relaxation with reappraisal with goals of both calming the physiological state and changing cognitive representations. Taken together, Linehan offers a well-structured rationale for integrating perceived physiological experiences into BPD treatment.

#### Support for (psycho)physiological assumptions of DBT?

The support for the underlying Biosocial Theory is, however, divided. On the one hand, there is support for the biosocial theory in self-report measures. For example, individuals with BPD report higher levels of distress during baseline assessments (63, 64) and slightly higher on self-report measures of hyperreactivity (65–67). Patients with BPD report particularly heightened reactivity to negatively valenced stimuli that are related to the disorder, such as abandonment or rejection (68, 69). Conversely, findings from physiological studies have not supported the biosocial theory (67, 70). A review by Cavazzi and Becerra revealed inconclusive results for both heightened emotional baseline and emotional reactivity in physiological measures (70). In fact, patients with BPD reported significantly lower HR, RSA, and blood pressure during baseline compared to healthy controls. The results on emotional hyperreactivity were equally unclear, with a trend in which individuals with BPD tend to react slightly stronger to negatively valenced stimuli. In other words, patients with BPD did not significantly differ from healthy controls in terms of their baseline arousal or reactivity to stressors. Another meta-analysis, based on 31 laboratory studies, also failed to provide support for the hyperreactivity hypothesis (67). In light of these findings, it can be concluded that patients with BPD may subjectively report more distress on BPD-specific stimuli (abandonment, interpersonal difficulties, social rejection), yet do not show physiological divergence in baseline and reactivity. This suggests that there may be a difference in the experience and interpretation of these stimuli, rather than a difference in bodily signals. This compelling evidence against the biosocial theory requires attention for future investigation or a potential revision of the theory. Recent articles aim for other operationalizations of Linehan’s first assumption of hypersensitivity. Instead of framing it as a higher baseline of physiological arousal, some studies test the possibility that it may be linked to a higher probability to experience stimuli as emotional (71, 72).

### Transference focused psychotherapy

#### Brief overview of the treatment and its underlying theory

TFP is an intensive twice-weekly individual psychodynamic treatment with its theory based upon the object relation theory. Kernberg’s object relation theory posits that human drives and needs are always experienced in relation to significant others (‘objects’) (73). Object relations are conceptualized as motivational structures that guides perception. These internalized relationships are assumed to affect all later relationships, motivations, and attitudes (73). According to Kernberg, patients with BPD have poor object relations and a chaotic internal structure, which translates to splitting defenses and polarized representations of self and others (74). In TFP, therapist start by setting a treatment contract with the patient, with goals to limit maladaptive coping strategies and ensuring the possibility of change. Subsequently, sessions are generally unstructured in nature, signifying that patients determine the subject to be discussed during their sessions. The therapist typically refers to the transference within the therapeutic context and uses countertransference as a vehicle for understanding the dynamics that unfold during treatment. The duration of TFP is typically a minimum of one year, though more commonly extends over several years. Interventions are similar to classic psychoanalytic interventions and include exploration, confrontation and interpretation, with the ultimate goal to promote emotion regulation and identity integration.

#### Utilization of physiology in treatment model

According to the treatment manual of TFP, little attention is directed towards interoception or physiology (36). In addition, they provide no interventions that relate to physiology or interoception and therefore provide little to no guidance for implementing biocueing. One might argue that psychoanalytic treatment in general, or working with transference in the therapeutic alliance, might boost someone’s awareness of physiological reactions or bodily reactions. After all, individuals during TFP treatment engage in mentalization and reflections on their emotional and bodily states. Nevertheless, TFP does provide little guidance for integrating biocueing technologies.

#### How does psychophysiological literature support TFP?

In addition, there is few literature that elaborates on the relationship between TFP and physiology. Unsurprisingly, the relationship between the object relation theory and physiology has not been studied, given that this theory is highly conceptual and lacks clear conceptualizations. It is only very recently that Clarkin et al., part of Kernberg research team, propose a theoretic model that demonstrates how individuals with BPD might display elevated autonomic responses due to negative appraisals (e.g. feeling threatened) on seemingly neutral social stimuli (75). In sum, TFP does not provide a theory or a therapeutic frame that facilitates the integration of biocueing technologies.

### Schema therapy

#### Brief overview of the treatment and its underlying theory

Schema Therapy is an integrative treatment with roots in cognitive behavior therapy, attachment, gestalt therapy, and emotion-focused traditions (37). The goal of the treatment is to recognize and ultimately reorganize patients’ inner structures. According to ST, borderline personality disorders emerge during childhood when emotional needs are insufficiently met, resulting in maladaptive cognitive beliefs of themselves, others, and the world. ST is traditionally delivered in weekly individual sessions, but is recently often combined with a group treatment (82, 83). The duration of ST is typically between one to three years, depending on the patient’s individual needs and the severity of their symptomatology (37, 84). In ST, therapists takes the role of a ‘good parent’ with interventions ranging from cognitive reappraisals to experiential exercises such as chair work or imagery rescripting. Since the conception of ST, it has evolved considerably, in particular with the advancement of the modes model (85). This model is a separate theory within ST and refers to certain mental and emotional states in the present, such as a state in which one feels abandoned, lonely, and vulnerable (vulnerable child mode), or a sudden harsh stance towards oneself (punishing parent). However, a clear theoretical frame for this mode approach is still lacking (85, 86).

#### Utilization of physiology in treatment model

Although the practitioner’s guide of ST refers to physiological changes as a component in the development of schemes, they provide no further intervention or rationale on physiology or interoception. Yet, the treatment does raise awareness of emotional and bodily sensations. In ST experiential work, for instance, it is common to start by raising awareness of bodily sensations, mostly used as an access point towards childhood memories. Due to a lack of direct interventions on stress physiology or interoception, ST provides little guidance on integrating biocueing.

#### How does psycho(physiological) literature support ST?

Similarly, there are few studies on how physiology changes over the course of a ST. A few studies examined how physiology relates to imagery rescripting, an experiential technique used in ST to target adverse childhood memories, but found inconclusive results. Some studies (mainly focusing on anxiety disorders) found that imagery rescripting had beneficial effects on reducing HR or increasing HRV (87–89), while others did not discover any effects on physiological reactivity (90, 91). Recently, a theoretical article is published with aims to connect modes through the lens of the polyvagal theory (92) (for an overview of the theoretical foundations of the polyvagal theory, see **Box 2**). This is, however, preliminary and does not yet provide implications for clinical practice. In brief, ST does not provide a clear direction on how to integrate biocueing technologies in the treatment frame.

Box 2 **Polyvagal theory.**

The Polyvagal Theory (PVT) is a theory that primarily focuses on the ANS with roots in evolutional biology (15, 93–96). Over the years, the PVT has proven to be useful in clinical practice and has been linked to many different psychiatric disorders (97), including BPD (98). The PVT provides a generic framework for elaborating how the body reacts to stressors, (potential) traumatic experiences (a frequent comorbid symptom in BPD) and danger.

The theory has become increasingly popular as an underpinning theory for new psychotherapeutic treatments (such as the sensorimotor psychotherapy or somatic experiencing) and also seems to fit as an underpinning theory for creative arts and psychomotor therapies (CAPTs) (78). The Polyvagal theory entails the ‘nervus vagus’, a nerve that wanders towards all major organs in the body, and posits that it consists of three separate branches. When comfortable and feeling safe, there is mostly activity in the ventral parasympathetic branch, a calm state which promotes social engagement and allows for bodily processes such as ‘rest and digest’. When faced with stressful or traumatic events, the sympathetic branch mobilizes the body for defensive responses, such as fight/flight responses. Patients with BPD are assumed to experience seemingly neutral contact as threatening and are thus more likely to mobilize for defensive responses, as demonstrated in Austin, Riniolo & Porges (98). When faced with prolonged distress, the body can resort to a last line of defense via the dorsal parasympathetic branch, which includes ‘feigning death’ behaviors and tonic immobilization. These separate branches are hierarchically embedded in our ANS.

In addition, PVT further coins the concept ‘neuroception’, a neurological circuit that allows our bodies to register whether the environment is safe or dangerous. Neuroception trigger shifts in autonomic states without the requirement of conscious awareness (99). People that are raised in threatening conditions, as is often the case in individuals with BPD, are more likely to have difficulties with neuroception. Seemingly neutral social interactions are perceived as potentially stressful and dangerous (98) and trigger defensive responses with long-term risks for prolonged stress or allostatic load (100, 101)​.   The polyvagal theory is an inspiration for different (bodily-oriented) psychotherapeutic treatments including sensorimotor therapy (SM) (102), somatic experiencing and is used as a underpinning theory for CAPTs (78). Despite the growing popularity of the polyvagal theory, some scholars have raised concerns about the empirical basis of the theory (103–105).

### Mentalization based treatment

#### Brief overview of the treatment and its underlying theory

MBT is an intensive psychodynamic treatment that usually combines group sessions with individual sessions on a weekly basis. The primary goal is to improve mentalization, a capacity to make sense of oneself and others on the basis of intentional mental states, feelings and beliefs (35). According to Fonagy and Bateman, BPD is in essence a disorder to accurately mentalize within interpersonal relationships, resulting in a broad range of BPD symptoms (106). MBT is rooted upon attachment theory (107, 108), theorizing how a lack of mentalization emerges during childhood in which caregivers may have mirrored inadequately (e.g. caregivers that cry in response to a tearful child), maltreated or neglected the child (109, 110). These experiences during early childhood result in an insecure attachments style or a false sense of self. Key interventions in MBT are the not-knowing stance, repeated repetitions of mentalization during therapy sessions, support and validation, and the repairment of ruptures in the therapeutic alliance. MBT is a process oriented treatment with the ultimate goal to slowly expand patients’ mentalizing capacities over several years (111).

#### Utilization of physiology in their treatment model?

Overall, MBT provide little attention on specific physiological processes in their treatment model and handbook, despite some references to the HPA-axis and physiological processes during childhood (109). MBT does, however, focus on managing the arousal levels of patients (not specifically targeting the physiological level but rather generally), ensuring that patients can remain engaged in the mentalizing process. Moreover, in the 4^th^ module (mentalizing emotions) of the handbook, they briefly list different techniques that can be helpful in managing hyperarousal within the body: progressive muscle relaxation, breathing skills, silence and meditation stance and mindfulness. There are no instructions on how to perform or incorporate these interventions in the therapy. The management of arousal levels is mostly discussed in a holistic manner with little specific focus on physiological processes. Nonetheless, it could be argued that physiological processes are already incorporated in the treatment, as the mentalization of emotions inherently includes the mentalization of bodily sensations, providing that emotions are embodied experiences (112). Yet, the MBT framework lack a clear trajectory on how to approach physiological reactions or how to utilize physiological reactions in tailoring helpful interventions. Recently, a movement within the MBT developed a framework for embodied mentalization (113–115). This is particularly integrated in treatments for patients with severe somatoform disorders (116). Fotopoulou and colleagues offer a valuable theoretical perspective on embodied mentalization, highlighting the importance of interoception as an implicit mechanism that provides information on how one fares in life (115). The movement of embodied mentalization is much broader than just physiological processes (e.g. it also entails gestures, body positions or facial expressions) and does not yet provide guidance for integrating technologies.

#### How does psycho(physiological) literature support MBT?

To our knowledge, few studies have included measuring physiology during a MBT treatment or the process of mentalization. Some authors claim that bodily awareness and interoception (in general) are a prerequisite for mentalization, mostly from a theoretical perspective (e.g. 114, 117, 118). Independent of the MBT framework, a line of research has sought to understand the physiological underpinnings of the attachment theory. This line of research, however, is broad and became a pillar for various psychotherapeutic treatments, including MBT, ST and TFP. For clarity, we elaborate on the attachment theory in a separate paragraph.

#### How does psycho(physiological) literature support attachment theory?

Attachment is an underlying biopsychosocial theory that is often brought up in patients with BPD. Many symptoms of BPD, such as the fear of abandonment or difficulties with interpersonal relations, are closely interrelated to attachment processes. This is unsurprising, given that individuals with BPD frequently report adverse childhood experiences and more often grow up in an affectively poor climate, thus developing more insecure attachment styles. Studies indeed indicated that a safe attachment style is found in approximately 0-8% of patients with BPD compared to approximately 59% in healthy controls (119, 120).

The process of attachment starts directly after birth, via both co-regulation processes and emotional connection with their parental figures (121, 122). Attachment is primarily an embodied process, where parents assist their baby in maintaining homeostasis by feeding, nursing, gently stroking, and providing skin-to-skin contact. It is crucial for newborns to feel safe in their environment, to self-regulate and engage in the bonding procedure. For instance, a study revealed that skin-to-skin contact with parental figures during the first hours of life favors better regulation in the HPA-axis in children (123). With sufficient care and repetition, referred to by Winnicot as *‘good enough’* parenting (124), a secure attachment style develops with matching calm physiological values (125, 126). However, when bonding fails, an insecure attachment style develops with matching alterations in physiology (127). The development of physiology and attachment in the early years of childhood is widely established during the strange situation test and is currently well understood. In this procedure, the child’s emotional, physiological and behavioral reactions are observed during a period of playing, separation and reunion with the mother. A meta-analytic review focusing on the ‘strange situation test’ reveals that children with insecure attachment styles show elevated physiological values (respiratory sinus arrhythmia (RSA) and cortisol) during the separation and recovery phase compared to securely attached children (127). This indicates that, although insecurely attached children report no baseline differences, they are more prone to interpersonal stress and less effective in emotion regulation (127).

Although similar results have been found regarding the relationship of attachment and physiology at adolescence and adulthood, the amount of evidence in adulthood is sparce. Attachment in adulthood is most reliably measured using the adult attachment interview (AAI) (128). Initial studies found that adults with secure attachment styles typically exhibit a lower HR response when discussing attachment-related topics (129) and show for lower levels of Skin Conductance Levels (SCL) compared to insecure attachment styles (129, 130). At first, studies proclaimed that heightened SCL were mostly present in dismissive insecure attachment styles, and attributed this as a sign of emotional suppression (129–131). Later studies found evidence that insecure attachment styles more in general were correlated with higher SCL levels (132–134). In a study on adults with a disorganized attachment style, a surprising result was found in which disorganized attached adults showed an increase in vagally-mediated heart rate variability (vmHRV) during the stress task (indicative of an increase of parasympathetic activity), which persisted during the recovery phase (135). The authors attribute this finding to an attempt at emotion regulation. Additionally, this study could not find any significant differences in vmHRV between secure and insecure attachment styles.

In sum, it is likely that a secure attachment results in overall calmer physiological values, which last towards adulthood. However, more research is needed, particularly on the relationship between physiology and attachment in adolescence and adulthood.

## Discussion

This narrative review examines the emerging field of biocueing technology and its potential applications in the treatment of BPD. Wearable technologies, such as smartwatches, smart clothing and smart rings, offer opportunities for real-time monitoring of physiological data, providing immediate feedback that could enhance emotion regulation strategies (2). The concept of biocueing, where individuals receive cues about significant physiological changes, presents an innovative biofeedback approach to aid patients with BPD in managing severe stress and emotional dysregulation (2).

Among the four psychotherapeutic evidence-based treatments for BPD — Dialectical Behavior Therapy (34), Transference Focused Psychotherapy (36), Schema Therapy (37), and Mentalization Based Treatment (35)— DBT stands out for its explicit integration of physiology in their treatment frame and interventions (34). DBT employs various interventions where biocueing could be fitted in, including the addition of objective data to physiological self-monitoring, as a tool for measuring the efficacy of mindfulness and relaxation exercises, to enhance bodily awareness and regulate autonomic nervous system responses. Particularly the TIPP interventions, such as prolonged relaxation or intensive exercises, could be addressed as candidates for just-in-time adaptive interventions whenever one’s autonomic nervous system may be dysregulated. Besides DBT, it could be promising to explore if biocueing technologies could be fitted within CAPTs, given their innate approach working from bodily sensations. The other evidence-based psychotherapeutic treatments for BPD largely disregard physiology in their treatment frame and use the attachment theory as their underpinning theory, with a treatment focus on relational and/or cognitive aspects (35–37). In any case, this illustrates that physiology is currently not actively engaged in most evidence-based psychotherapeutic treatments (it might be targeted more implicitly). This does not automatically imply that these treatments could not benefit from certain interventions or technologies. For instance, for TFP, could HRV be a measure of the therapeutic alliance (e.g. high HRV signifying a positive therapeutic alliance), as referred to by Dufey et al. (136). For ST, it may be interesting to explore whether certain modes (more frequently) relate to certain physiological states. For instance, does the detached protector mode relate to a lower HR/HRV/breathing rate (indicative of increased parasympathetic activity) and/or heightened SCL (indicative of increased sympathetic activity and suppression). As for MBT, could monitoring objective physiological data assist therapists in the management of patients’ arousal levels. Finally, it could be interesting to explore new avenues and directions that could improve treatments overall, rather than merely aligning it. For instance, it could be worthwhile to explore whether general stress monitoring contributes to therapy among individuals with BPD.

Two major theories, namely the biosocial theory and attachment theory, are frequently referred to as underpinning theories of evidence-based treatments for BPD. Interestingly, these theories receive inconclusive to mixed support from psychophysiological studies (67, 70, 127). Specifically, the biosocial theory yields mixed to no support in physiological studies (67, 70), whereas physiological studies on attachment theory in adulthood have identified a correlation, but remain inconclusive due to the low number of studies (129–135). Already, this discrepancy in support between the two theories is somewhat unexpected, given the partial overlap among patients with BPD between these theories. Although the biosocial theory mainly focuses on patients with BPD, the majority of individuals with BPD are known to report insecure attachment styles. As stated before, previous research has indicated that only 0-8% of patients with BPD report a secure attachment style (119, 120, 137). Accordingly, one would expect that studies on the biosocial theory would yield somewhat similar results to those of attachment theory. It is plausible that this discrepancy in results is due in part to differences in methods. Firstly, studies of the biosocial theory typically manipulate stress with impersonal tasks, such as viewing BPD-related videos or pictures, or participating in the Trier Social Stress Task (67, 70). In contrast, adult attachment research typically involves the AAI, an interview that requires the recall of childhood memories that are highly personal and, potentially, traumatic. Secondly, there is also a notable difference in respondents. Research in biosocial theory focuses primarily on borderline pathology whereas the field of attachment theory is not limited to categorical diagnoses. Thirdly, it is also possible that research on attachment in adulthood is underpowered. In any case, as insecure attachment styles are correlated to heightened physiological reactions, it could be a promising target for biocueing monitoring and interventions.

In light of the latest psychophysiological insights and from a psychophysiological perspective, one might question the continued validity of biosocial theory. A major challenge is its underlying one-to-one assumption, in which BPD pathology corresponds to certain physiological alterations. Similar to Siegel’s critique on the one-on-one “emotional fingerprint” hypothesis (21), one could argue whether such a correspondence exists, especially considering the heterogeneity of BPD. A many-to-many correspondence is more likely, where there is a high variability between individuals and a high variability in ANS reactivity. Furthermore, previous studies suggest that ANS variability is further dependent on interactions with its environment as well (138, 139). The biosocial theory currently does not take environmental factors into account. Taken together, these results may not be satisfactory, as they are not in line with the current rationale of the biosocial theory and are challenging to integrate.

### Interoceptive magnitude as a gateway to emotional processing

**Figure 1:**
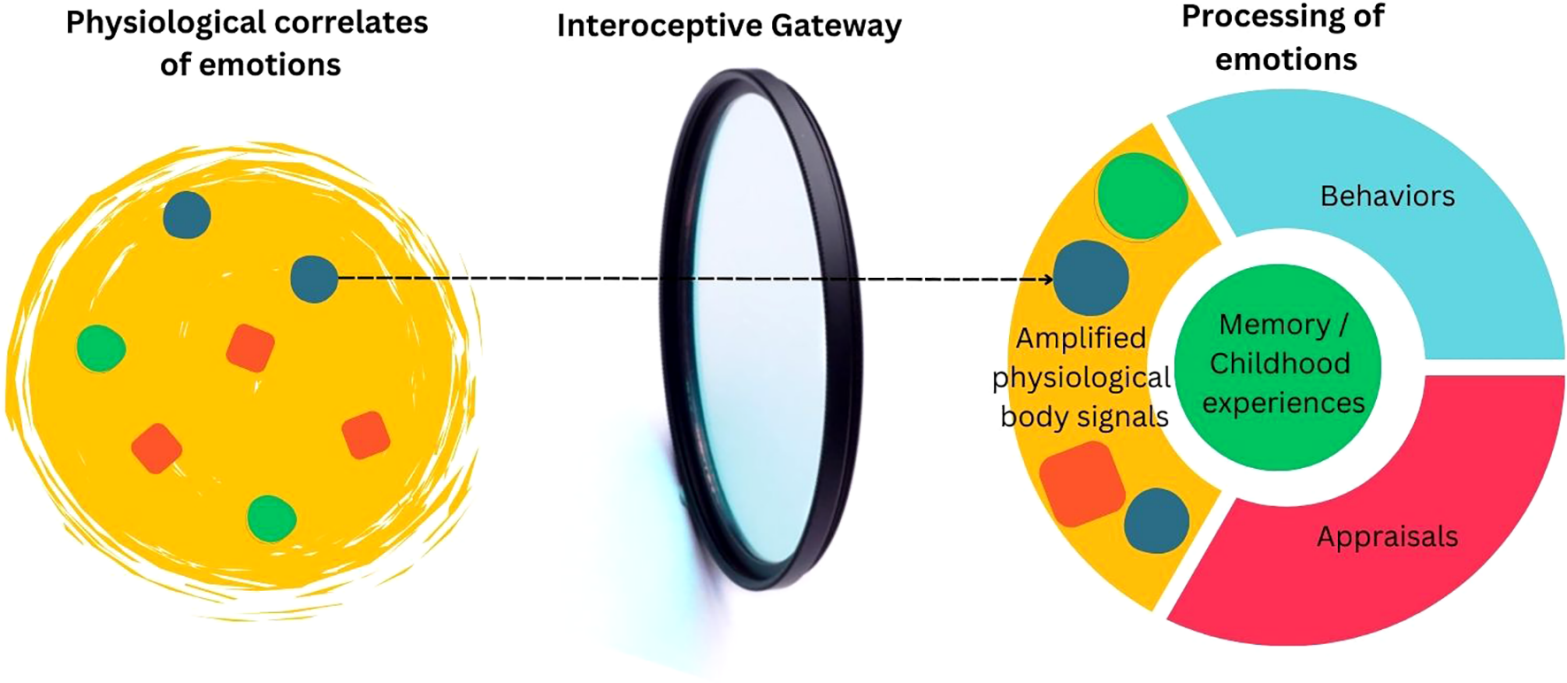
Example of an amplified interoceptive gateway.

It is compelling that individuals with BPD do not differ significantly from healthy controls in physiological reactions (67, 70), yet do (self-)report higher levels of emotional arousal (65, 66, 140, 141). It is possible that this contrast may be explained by other ANS measures that were not included in these study designs (e.g. muscle tension). Another explanation may lie in a different interoceptive processing of similar bodily sensations. It is interesting to consider if there may be a metaphorical gateway that shapes *how* physiological correlates of emotions are processed. Some individuals, such as those with BPD, may possess an amplified gateway, analogous to a magnifying glass, through which bodily signals are more quickly experienced as intense or overwhelming (see **Figure 1**). In contrast, other individuals may have a diminished gateway, which impairs their ability to derive meaning from bodily sensations and which approach emotions and experiences more rationally.

In terms of the interoceptive literature, it remains quite unclear how to operationalize this, especially in the light of recent developments within field of interoception research (see **Box 3**). For instance, this gateway could be due to a difference in for instance interoceptive attention or interoceptive magnitude (self-perceived intensity of bodily signals). From a theoretical perspective, it could be argued that alterations in both interoceptive attention and interoceptive magnitude may be expected to develop during childhood. To illustrate, for children who are raised in traumatic or abusive environments, it may be beneficial to disregard bodily sensations, become attentively focused on external cues and become easily alarmed as an adaptive response to a maladaptive and threatening environment. In this case, one might expect an interoceptive blueprint with reduced interoceptive attention and amplified interoceptive magnitude, thereby enabling a rapid reaction when faced with a threat. Following Fotopoulou et al, interoception may be embodied processes inherently reflecting beliefs and experiences of one’s childhood (115). Finally, this hypothesis could potentially also align with the newer operationalization of Linehan’s hypersensitivity, in which individuals with this heightened interoceptive magnitude are expected to have a higher probability of experiencing intense emotions (34, 71, 72).

Box 3 **Recent developments in the field of interoception.**

As mentioned previously, the concept of interoception has historically been divided into three distinct measures: *interoceptive accuracy* (objective perception of internal signals), *interoceptive sensibility* (self-perceived perception of internal signals, e.g. questionnaires), and *interoceptive awareness* (metacognitive awareness of interoceptive accuracy) (49). However, recent reviews highlight significant concerns on the construct validity and measurement of these constructs (48, 142, 143). For instance, Desmedt illustrates that interoceptive accuracy in one system (e.g. cardiovascular) is often not predictive of interoceptive accuracy through another system (e.g. respiratory)” (48). While this on itself is already quite concerning for the construct validity, moreover, a recent meta-analysis reported equally low convergence between different interoceptive accuracy measures within the same bodily system (48, 144). These findings highlight the necessity for a revised theoretical framework of interoception, along with the development of new methods to measure interoceptive processes.

Recent studies propose comprehensive frameworks that extend the number of factors involved in interoception. Khalsa et al., propose a framework that divides interoception into six distinct measures: interoceptive attention, -detection, -discrimination, -intensity, -accuracy and -self-report (145). Desmedt et al. present a framework with four overarching factors (interoceptive attention, -sensing, -interpretation and -memory) and 11 subfactors (48, 146). It is imperative that future research elucidates which conceptualisation is the most appropriate and which measures most accurately capture these constructs. While such developments are integral to the field’s progression, the current coexistence of multiple frameworks may hinder conceptual clarity and consistency in research.

### Future directions of biocueing

The incorporation of psychophysiological inferencing into treatment models for borderline personality disorder presents several challenges. Firstly, our understanding of psychophysiology is largely based upon laboratory studies, wherein the environment is highly controllable and predictable (147). In contrast, ambulatory measures are inherently uncontrollable and result in greater variability, both in terms of (psycho)physiology and stressors from the environment (10, 138, 139). Secondly, there are major challenges in how to embed information concerning the context into technologies. Thirdly, many biocueing technologies focus on one or two physiological signals, which provide some information of the ANS, but certainly not all. In the future, it could be imaginable to combine multiple sensors within one wearable to unlock the full potential of biocueing technologies. Fourthly, while substantial variability exists between individuals, emerging evidence suggests that there is greater coherence in levels of emotional arousal within individuals [e.g. (148)]. This implies a need for idiographic studies that examine how physiology behaves within one individual that is willing to be investigated for longer periods.

Thus, a major challenge in proceeding with biocueing technology resides in the synthesis of technology, physiology, psychology, and the environment. It highly depends on the intended purpose and users to consider which aspects of physiology, psychology and environment are deemed important. We foresee four potential non-exclusive avenues:

1. Focus on psychological processes and behavior change: largely ignore physiological processes and biocueing technologies in psychotherapeutic treatments.
2. Mainly focus on physiological data for monitoring long-term health gains.
3. Develop complex algorithms capable of embedding the environment by processing combinations of data.
4. Keep a human in the loop to evaluate for the environment.

A first avenue would simply neglect physiology in psychotherapeutic treatments and would not consider implementing biocueing interventions. Although this does not sound appealing at first, one conclusion might be that linking specific physiological processes to psychological processes within a clinical framework is not supported by sufficiently empirically validated scientific models. For instance, one could argue to what extent objective measures of physiological processes actually relate to subjective experiences (149). Individuals may describe conscious or schematic representations of bodily states, rather than actual objectifiable bodily states. To illustrate this point, it is possible that someone subjectively experiences high severity of stress, whereas different sensors may display normal or regular values. Specifically, this approach abandons biocueing technologies for psychotherapy for the time being and focuses on psychological processes.

A second avenue mainly focuses on physiological processes by utilizing biocueing technologies to monitor and detect for risks of biological diseases. This approach is equally skeptical that short-term physiological processes can be meaningfully linked to psychological processes. Instead, it focuses on monitoring and long-term health benefits of wearing sensors. Some studies, for instance, examined the relationship between HRV as a general indicator of psychiatric disorder severity or as a long-term measurement of general health (136). From this perspective, physiological data are primarily used to optimize long-term health outcomes or monitoring progress, reducing the immediacy or necessity of biocueing for real-time feedback. In such cases, continuous remote monitoring by healthcare professionals can replace the need for client-centered biocueing, allowing professionals and AI to track risk factors and intervene when necessary, without requiring the patient to actively engage with their physiological signals on a daily basis. This approach prioritizes the cumulative assessment of long-term risk factors for physical and mental health over the potential for short-term psychological insight or behavior change (e.g 150). However, a recent realist review suggests that remote measurement technologies for mental health, such as biocueing devices, may be most useful in a direct role (151). These technologies have been shown to enhance emotional self-awareness and facilitate a stronger therapeutic relationship through real-time feedback, which can contribute to better symptom management and encourage help-seeking behaviors, especially in young individuals with depression (151).

A third avenue also integrates physiological, psychological processes with environmental factors from a technological perspective. This avenue explores embedding the environment by processing multiple streams of data with the help of machine learning (e.g 152, 153). This field is known as digital phenotyping, referring to the digital footprint left behind by patient-environment interactions (154). In practice, this could involve using a wearable device that not only monitors HRV but also tracks physical activity, GPS data, sleep patterns, and even subjective self-reports of mood and stress levels through a mobile app (154). For instance, when the device detects significant reduction in HRV (indicative of lower parasympathetic activity), it would consider the context—such as whether the individual is at work, at home, or in a social setting—before providing a tailored cue. If the individual is at work and reports feeling overwhelmed, the JITAI (just-in-time adaptive intervention) might suggest a short mindfulness exercise or a brief walk outside. If the same HRV change occurs at home during the night, the JITAI intervention might instead recommend a relaxing activity or a mindfulness exercise. This approach equally acknowledges the complexity of emotional and physiological responses and leverages multiple data points to provide more accurate and personalized support, enhancing the effectiveness of biocueing in real-world settings. A major downside of this approach is its intrusiveness (152, 154). This type of biocueing technologies presents significant privacy and ethical considerations that must be carefully considered. For instance, it raises ethical questions about the extent to which technologies should (or should not) interfere in people’s lives. Additionally, the processing of vast quantities of data gives rise to privacy concerns that may potentially contravene current legislation, such as the European AI Act. In order to proceed, this approach requires a broad collaboration of engineers, scientists, clinicians, and patients in order to develop technologies that fit the targeted population.

A fourth avenue aims to integrate physiological, psychological processes and the environment by developing technologies that maintain human involvement in the process. The technology does not interpret but simply prompts users to reflect and assess the environment upon receiving a biocue. This type of technology can be used as a preventive tool (e.g. using biocues to reduce aggression behaviors (155)), as a coaching tool (e.g. using biocues to foster interoceptive attention/detection), or as reference tool (e.g. to highlight important events during therapy) (156). While these prompts may resemble Just-In-Time Adaptive Interventions (JITAI), they diverge from standard implementation. In a classic JITAI framework, support is tailored and delivered automatically by the system in response to dynamic internal or contextual states, following pre-specified decision rules and tailoring variables designed to optimize timing and type of support. In contrast, the current approach avoids fully automated adaptations, favoring user-led reflection over system-determined actions (157). In favor of this approach is also its acknowledgment of the complex nature of the autonomic nervous system and psychological experience, thereby reducing the likelihood of overinterpretation of physiological data as cautioned by de Geus and Gevonden (10). As a potential downside of this approach, it remains unclear if these biocues are relevant and provide any gains for the individual. Thus, future research could use idiographic studies to shed light on the added value of biocueing and explore which aspects of interoception (e.g. interoceptive magnitude, interoceptive detection or interoceptive attention) are potentially targeted by biocueing. Nevertheless, the non-invasive, compassionate and gentle approach of these technologies makes them promising for use in the near future, as these technologies are already available and can be fitted alongside existing psychotherapeutic treatments, including DBT, MBT, ST, CAPTs and potentially TFP.

## Strengths and limitations

This review should be regarded in considerations of its key strengths and limitations. A notable strength of this article is its pioneering effort to explore the intersection of physiology, psychology and biocueing technologies within the field of borderline personality disorders. This review provides a state-of-the-art overview of recent biocueing technologies and its potentials within the field of personality disorders and their underpinning theories. It highlights gaps in our knowledge, making it a valuable contribution to the field. However, this narrative review has also several limitations that must be acknowledged. Most importantly, the absence of a systematic literature search may have led to an unintentional selection bias and an incomplete representation of the existing evidence, potentially affecting the comprehensiveness and generalizability of the conclusions. Secondly, the often not specified way physiology is attended to in therapy can be problematic. For instance, it is common practice in most treatments for personality disorders to monitor patients’ arousal, co-regulate their emotions and watch for potential ruptures in the alliance. The extent to which professionals specifically attend to physiological changes in patients and how they intervene on physiological arousal often remains highly unclear. To address this issue, this review focused only on explicit physiological processes and thereby disregarding potential implicit ways in which physiological activity may have been attended to. However, by doing so, there is an emphasis on objective physiological data (e.g. HR or HRV) rather than subjective perception of bodily sensations. As a result, subjective perceptions of physiological processes may be underrepresented.

## Conclusion

To conclude, the landscape of psychophysiology is complex and faces severe challenges in incorporating physiological data in treatments for borderline personality disorder. Physiological theory is neglected in most psychotherapeutic treatments (except for DBT/CAPTs), potentially because of the intricate interplays between physiology, psychological factors, environmental factors, and subjective experiences. The incorporation of biocueing technologies in treatments for borderline personality disorder is challenging but offers exciting opportunities to improve the mental health of individuals with borderline personality disorder. Interdisciplinary collaboration between engineers, researchers, and clinicians is essential for developing and refining biocueing technologies, ensuring they are both effective and safe for clinical use. Four avenues for future research are proposed: abandoning physiological data, biocueing devices as long-term (mental) health monitors, machine-learning biocueing interventions, or biocueing while keeping a human in the loop. The latter may be the most promising for the near future to explore if interoception, and specifically which interoceptive processes, can be boosted by biocueing.
